# 1856–1889

**Published:** 1889-01

**Authors:** 


### 1856-1889.

Since October 1856 the present editor of the DENTAL REGISTER has been continuously in this service, never having during this thirty-two years and more, omitted a single number of its publication, and almost every number has had the personal supervision of the editor. The first four years, or up to July 1860, it was issued quarterly, from that to the present time it has been published monthly. Up to vol. 25, 1871, it was edited jointly by J. Taft and Geo. Watt. Since that time the present incumbent has been the sole editor. The REGISTER was first issued in 1847, and is now in its forty-third year, being antedated only by the *American Journal of Dental Science*. The work of this thirty-two years has been accomplished under some embarrassments; during all of this time a full professional practice required and received attention; and in addition educational work in the way of teaching, etc., has demanded a large portion of the time of each year; and in addition to these other labors for the profession have seemed necessary and could not well be neglected.

Now no one knows better than the writer that all this is more than ought to be undertaken by one person; but much of it was taken up under protest, and with the expectation that a prolonged service would not be required, but generally such expectation has not been realized.

The progress and changes that have been made in the science and art of dentistry in this third of a century, is incalculable. The student of the history of the profession will perceive in it a prophecy for the future. Doubtless with all the agencies now in operation, progress in the coming years will be far greater than in the past; and may each and every one see to it that his part in the great work is accomplished.

